# Sexual and Metabolic Differences in Hippocampal Evolution: Alzheimer’s Disease Implications

**DOI:** 10.3390/life14121547

**Published:** 2024-11-26

**Authors:** José Manuel Martínez-Martos, Vanesa Cantón-Habas, Manuel Rich-Ruíz, María José Reyes-Medina, María Jesús Ramírez-Expósito, María del Pilar Carrera-González

**Affiliations:** 1 Experimental and Clinical Physiopathology Research Group CTS-1039, Department of Health Sciences, Faculty of Health Sciences, University of Jaen, Las Lagunillas University Campus, 23009 Jaen, Spain; jmmartos@ujaen.es (J.M.M.-M.); mramirez@ujaen.es (M.J.R.-E.); 2 Department of Nursing, Pharmacology and Physiotherapy, Faculty of Medicine and Nursing, University of Córdoba, 14004 Córdoba, Spain; n92cahav@uco.es (V.C.-H.); en1rirum@uco.es (M.R.-R.); cm2remem@uco.es (M.J.R.-M.); 3 Maimonides Institute of Biomedical Research of Córdoba (IMIBIC) IMIBIC Building, Reina Sofia University Hospital, Av. Menéndez Pidal, s/n, 14004 Cordoba, Spain

**Keywords:** hippocampus, Alzheimer’s disease, glucose transporters, GLUT4, renin–angiotensin system, insulin regulated aminopeptidase, sex, brain

## Abstract

Sex differences in brain metabolism and their relationship to neurodegenerative diseases like Alzheimer’s are an important emerging topic in neuroscience. Intrinsic anatomic and metabolic differences related to male and female physiology have been described, underscoring the importance of considering biological sex in studying brain metabolism and associated pathologies. The hippocampus is a key structure exhibiting sex differences in volume and connectivity. Adult neurogenesis in the dentate gyrus, dendritic spine density, and electrophysiological plasticity contribute to the hippocampus’ remarkable plasticity. Glucose transporters GLUT3 and GLUT4 are expressed in human hippocampal neurons, with proper glucose metabolism being crucial for learning and memory. Sex hormones play a major role, with the aromatase enzyme that generates estradiol increasing in neurons and astrocytes as an endogenous neuroprotective mechanism. Inhibition of aromatase increases gliosis and neurodegeneration after brain injury. Genetic variants of aromatase may confer higher Alzheimer’s risk. Estrogen replacement therapy in postmenopausal women prevents hippocampal hypometabolism and preserves memory. Insulin is also a key regulator of hippocampal glucose metabolism and cognitive processes. Dysregulation of the insulin-sensitive glucose transporter GLUT4 may explain the comorbidity between type II diabetes and Alzheimer’s. GLUT4 colocalizes with the insulin-regulated aminopeptidase IRAP in neuronal vesicles, suggesting an activity-dependent glucose uptake mechanism. Sex differences in brain metabolism are an important factor in understanding neurodegenerative diseases, and future research must elucidate the underlying mechanisms and potential therapeutic implications of these differences.

## 1. Introduction

The demographic aging associated with an improvement in well-being has led to an increase in the population over 65 years of age, carrying in parallel the increase in chronic pathologies associated with age. However, in the case of neurodegenerative diseases such as Alzheimer’s or Parkinson’s, we do not have sufficient knowledge about the initial causes for their approach and consequently for effective treatment, beyond the treatment of the associated clinical symptoms [1].

Neurodegenerative diseases currently represent a silent pandemic and a real challenge for the scientific community since, while we know different modifiable factors associated with their appearance, we also know other non-modifiable factors such as sex or age. During aging, the brain undergoes alterations at the level of energy metabolism, mainly related to glucose intake or insulin signaling, altering balances between routes. However, this circumstance is not comparable to that which could occur in the case of AD, where the described glucose hypometabolism occurs before clinical symptoms begin to manifest. In both circumstances, sexual differences have been described [1].

Within the older population, differences regarding sex in the prevalence of Alzheimer’s disease (sporadic or non-genetic cause) and Parkinson’s are evident, the first being mainly associated with the female sex, especially after menopause, and in the case of Parkinson’s disease, it is related to the male sex. In fact, both in Alzheimer’s disease (AD) and depression, women show a higher prevalence of the disease and a greater deterioration of memory or more severe cognitive symptoms in both disorders [2,3,4]. While, in the case of males with Parkinson’s disease, they present greater cognitive alterations than women with this same disease [5,6].

Therefore, sex hormones could have an important role in the modelling of the structure and plasticity of the hippocampus, a brain structure linked to cognition, which can contribute to greater vulnerability within each sex by type of disease. This evidence makes us think that evolutionary, anatomophysiological, and metabolic differences have been determined primarily by the hormonal profile of the female and male sex, as well as by the synthesis of endogenous cerebral estradiol, especially in the context of cognition and neuroplasticity, which could shed light on the differences in the mentioned neurodegenerative pathologies.

## 2. Evolutionary Characteristics in the Human Brain

The brain, apart from being an organ governing the most fine and precise bodily functions, is also the central component of human identity, so understanding this organ is essential to understand why we are what we are. The human lineage appears to have arisen from changes in neuronal connectivity, where slight changes in the connectome could lead to deep and specific functional changes. The connectome is made up of countless types of neuronal cells and their specific synaptic connections that make up the central components of circuits and neural networks [7]. The latest advances in imaging technology show the immense complexity of the human connectome, leading us to understand that our understanding of its organization and function at the level of long-range projections, local synaptic circuits, and intracellular signaling is still incomplete. In fact, the latest estimates suggest that there may be between several hundred trillion and more than a quadrillion synapses in the human CNS [8], with an average of 164 trillion synapses in the neocortex of young adult men [9]. On the other hand, the white matter of young adults contains between 149,000 and 176,000 km of myelinated axons [10], which can give rise to hundreds of thousands of distinct long-range projection systems [11]. Within its enormous complexity, the topology and stability/reliability of the connectome are fundamental for the establishment of the patterns of dynamic activity that underlie the cognition and specific behavior of each species [7,12,13]. In fact, the peculiarities of the human connectome appear to be unique in the pattern of neocortical myelination, which may have implications for the speed of conduction along the axons [14,15,16]. If we compare the human brain with that of other primates such as the macaque, it has a larger and less myelinated total axon surface, which mainly represents the association areas [15], which points and reinforces the idea that humans have reorganized the corticopetal, intracortical, and long-range corticofugal projection systems, especially those associated with the prefrontal and temporal association cortices, which are regions involved in higher-order cognitive functions. Therefore, both local circuits and long-range networks have undergone structural, molecular, and functional reorganization during human evolution, and these characteristics may have evolved independently of the described increase in brain volume [17].

In this sense, and from an evolutionary point of view, the hippocampus would soon become a critical brain structure for decision making, determining the importance of incoming sensory signals. Once this critical decision-making capacity is established, the hippocampus would be in charge of decision making. That is, if the hippocampus indicates that a neuronal input is important, it is likely that the information will be stored in memory, consolidating long-term verbal or symbolic memories.

The hippocampus, and its adjacent structures of the temporal and parietal lobes, collectively called the hippocampal formation, has numerous connections, indirect with different regions of the cerebral cortex, as well as with the basal structures of the limbic system: the amygdala, the hypothalamus, the septum, and the mammillary bodies. Therefore, almost any type of sensory experience causes the activation of at least one part of the hippocampus, which in turn distributes many output signals to the anterior thalamus, the hypothalamus, and other parts of the limbic system, especially through the fornix, an important communication pathway. Thus, the hippocampus is a channel through which incoming sensory signals can initiate behavioral reactions for different purposes. In fact, the stimulation of different areas of the hippocampus can cause almost any of the different patterns of behavior, such as pleasure, anger, passivity, or excess sexual desire [18,19].

However, the increase in expansive connectivity and brain volume throughout evolution carries a concomitant problem, the metabolic cost. That is, generating and maintaining an increasingly complex connectome has a substantial metabolic cost, since the human brain uses 18% of the body’s oxygen at rest, but it represents only 2.5% of the total body weight of a human being [20]. This circumstance required that during human evolution, molecular adaptations were produced to maintain high levels of neuronal activity, along with changes in energy allocation and diet [21,22,23,24].

## 3. Energy Metabolism in the Human Brain

Energy metabolism is crucial for the evolution of the human brain. An adult human brain already requires nearly 20% of energy expenditure; this figure rises in the case of newborns, who require >50% of energy consumption for an approximate weight of 11% of body weight [25,26]. Therefore, the management and use of energy is a very important point in relation to brain size [27].

Taking the evolution of lipid metabolism as an example, metabolic evolution has occurred through several mechanisms, which have led to the metabolic state of current higher organisms, including humans. Firstly, specific steps of energy metabolism were concentrated in specialized tissues, such as the liver and adipose tissue. Secondly, hormonal and neurological control of cellular functions would develop, which would change gene expression and concentrations of signaling metabolites. A third mechanism is gene duplication, where a copy of a gene would be sufficient to carry out the original function, and a final mechanism, multifunctionality, that is, the use of an existing protein to perform a new role while still performing its original function [28].

However, in the brain there is no net intake of fatty acids [29], which is paradoxical given the high energy needs of the brain. It is clear that circulating fatty acids do not enter the brain and do not directly “feed” cerebral energy metabolism, but they do perform two important indirect functions in brain energy; covering a substantial fraction of the energy needs of organs other than the brain, thus saving glucose for use by the brain, and serving as a source of ketone bodies, which do enter the brain and provide an important source of energy during fasting [30,31,32]. In fact, body fat at birth provides insurance for the brain between intakes, as fuel and nutrient. In this way, in non-human primates and premature babies, who do not have body fat or have very little, this possibility is compromised [33]. Before birth, the deposition of fat in the human fetus represents 90% of its weight gain [34,35] and therefore is a protective mechanism of the high energy needs of the human brain after birth, having a large reserve of fat available to produce ketones. For this reason, in infants, blood ketones are always slightly elevated, regardless of feeding status, a circumstance that does not occur in fed adults. In fact, in human fetuses at mid-gestation, ketones are not only an alternative fuel, but they appear to be an essential fuel, as they provide up to 30% of the energy the brain needs at that age [36].

However, in adults, glucose is the main fuel for the brain. The brain can oxidize ketones, but it does not oxidize the fatty acids from which they come. If food is restricted, the body’s glucose reserves (glycogen) last less than 24 h. and without ketones, brain function would be quickly compromised, or it would be necessary to degrade muscle protein to release amino acids that can be converted into glucose. Therefore, ketones would be an essential alternative fuel to glucose for the brain [37]. In fact, Cunnane and his colleagues have recently demonstrated that, while glucose metabolism in the brain decreases with normal aging and more severely in AD, the ability to metabolize ketone bodies, the other source of brain energy (during starvation or the ketogenic diet), remains normal in older people and patients with AD as their use depends on the plasma concentrations of the main ketones; acetoacetate and 3OH-hydroxybutyrate [38,39], according to data from studies of people following prolonged fasting regimens or a diet rich in ketone precursors, medium-chain triglycerides (MCT). In fact, some analyses, but of small sample size, in patients with AD or mild cognitive impairment show that metabolic interventions can improve cognitive processing [40,41]. These therapeutic strategies were recently termed “neuroketotherapeutics” and have shown some efficacy in preventing cognitive decline in people with AD [42].

Therefore, the cerebral metabolic rate of glucose is low during fetal development, increases linearly after birth, and reaches its maximum around 3 years of age, remains high during the first decade of life, and then gradually decreases during the second decade of life until it reaches the rate of glucose utilization observed in the early years of adulthood [43]. However, glucose needs to cross the BBB before entering the extracellular cerebral space. The glucose transporter, GLUT1, is the fundamental vehicle that facilitates the entry of glucose into the brain [44], which is regulated by 17 estradiol [45].

## 4. Sexual Differences in Brain Evolution Metabolic Differences

The brain of mammals is a sexually dimorphic organ. Throughout the evolutionary process, the human brain has undergone enlargement, which, on average, is greater in the case of men, with a total brain volume larger than women. This difference was also established in relation to certain brain regions; however, they were discarded when adjusted to the intracranial or total brain volume as a correction factor [46,47], approaching or equalling the volumes of specific structures in both sexes [7]. Studies in humans do show sex differences in relation to functional brain connectivity [48,49,50], as well as in relation to energy metabolism. Women have more interhemispheric connections compared to men, while men have strong intrahemispheric connections compared to women, and metabolic differences intrinsic to male and female physiology have also been described, therefore considering it fundamental to value biological sex in brain metabolism as well as in associated pathologies, such as Alzheimer’s disease, characterized by a higher prevalence in the female sex and cerebral glycosidic hypometabolism and a significant affectation of cognitive functions. In this context, it is important to point out the hippocampus [51]. In the structure of the hippocampus, where there are sex differences in relation to its volume, both at a regional level and in connectivity [52,53]. Although variations of it have not been described throughout development, we must take into account that there are variables, which perhaps are not always documented, that throughout life can affect its volume, such as early adversity [54], the phase of the menstrual cycle [55], the state of parity [56], hormonal therapy [57], the menopausal state [58], the genotype [59], and testosterone levels in men [60], and therefore these factors must also be contemplated to correctly understand the sexual differences of the hippocampus.

The enormous plasticity of the hippocampus is mainly due to the presence of adult neurogenesis in the dentate gyrus [61,62,63], fluctuations in the density of the dendritic column/synapses, dendritic arborization [64], and electrophysiological plasticity [65,66]. This plasticity is modified in a sex-dependent manner, either basally or by exposure to certain factors such as stress.

Hippocampal differences in relation to sex are linked to the presence of sex hormone receptors such as androgen receptors (AR) and estrogen receptors (ER): α, β, the G protein-coupled protein receptor (GPCR), as well as a high concentration of glucocorticoid and mineralocorticoid receptors compared to other regions of the brain, which makes the hippocampus more vulnerable to their ligands [67,68,69,70].

From a metabolic point of view, in the hippocampus, recent transcriptional studies [71] have described an enrichment of metabolic pathways in women compared to men, such as glycolysis or gluconeogenesis, purine metabolism, and the pyruvate pathway, while the metabolism of amino acids such as Ala/Asp/Glu, Arg biosynthesis, and glutaminergic synapses are decreased in healthy women compared to men. In addition, the TCA cycle, the hypoxia-inducible factor-1 (HIF-1) pathway, and insulin secretion were shown to be higher (more active) in women than in men. However, this relationship is nullified in the case of AD in women.

An increase in proteins involved in the transport and uptake of insulin-like growth factor (IGF) has also been described in healthy women compared to men, but in the case of Alzheimer’s disease, there is a decrease in proteins involved in the response to insulin stimulus and in the regulation of insulin secretion by GLP1 (glucagon-like peptide-1), some of which are involved in GABAergic synapses and circadian synchronization. In addition, proteomic data show a higher expression of insulin-like growth factor binding protein 7 (IGFBP7) in women with AD. The secretion of this protein is positively regulated in response to oxidative stress and is related to insulin resistance and the alteration of insulin signaling in AD, being a critical regulator of memory consolidation [72]. However, in the case of men, a significant difference has been observed in the expression of mitochondrial pyruvate transporter 1 (MPC1) in AD compared to healthy men. MPC1 appears to play a central role in glucose-stimulated insulin secretion, systemic glucose homeostasis in β cells, and the state of insulin resistance [73]. Therefore, these data would point to different pathophysiological mechanisms active in men and women in AD.

### Glucose Metabolism and Transporters in the Hippocampus

The glucose requirement for neuronal metabolism at the brain level is still unclear. This is mainly due to the transformation of glucose into lactate by astrocytes, which can be exported and used as fuel by neurons, and by its consumption by other cell types. However, neurons uptake and metabolize glucose through glycolysis as the main fuel for their normal function, with the proper maintenance of glucose metabolism being crucial to “sustain” learning and memory tasks, as its decrease leads to cognitive deficits [74,75]. This cellular glucose uptake process requires the presence of glucose transporters, as glucose is a polar molecule, insoluble in the plasma membrane, thus essential for glucose flux.

In human hippocampal neurons, both glucose transporters 3 and 4 have been described in axons, GLUT4 and GLUT8 in soma, and GLUT3 in dendrites [76,77]. GLUT3 is a low-capacity glucose transporter expressed in the plasma membrane of neuropil segments, ensuring basal support of glucose to neuronal activity. As for GLUT8, it has been described in hippocampal neurons, astrocytes, and microglia in murine models but intracellularly [74,78,79,80], initially not playing a significant role in cerebral glucose consumption, as there is no evidence of intracellular free glucose concentration, especially in neurons expressing GLUT4. GLUT8 is capable of translocating in response to insulin in the hippocampus, although despite responding to insulin in blastocysts, it has not been confirmed in neurons. Furthermore, GLUT8 shows a very low Km for glucose transport, compromising a potential significant role in hippocampal glucose metabolism.

**Figure 1:**
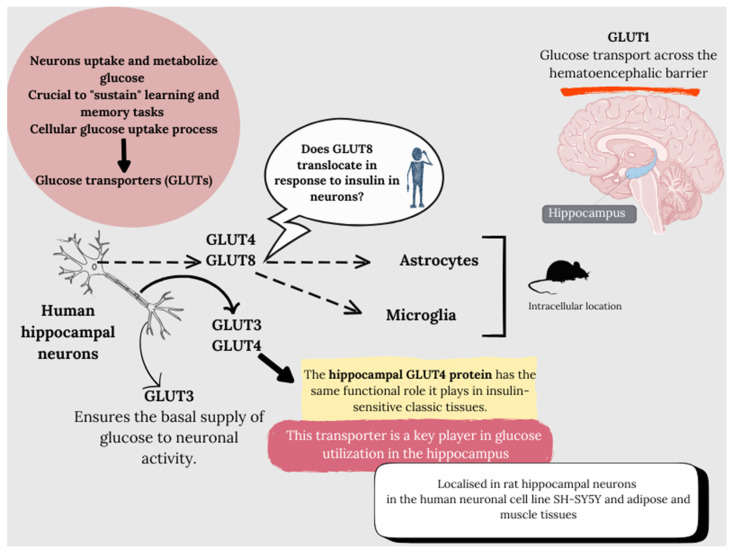
Glucose transporters in hippocampal tissue.

The medulla oblongata and hypothalamus were initially identified to have GLUT4 in the early 1990s, followed by the cerebellum and the hippocampus’s dentate gyrus. It was subsequently reported that the GLUT4 protein translocates to the plasma membrane in response to insulin, both in rat hippocampal neurons and in the human neuronal cell line SH-SY5Y, like adipose and muscle tissues. Additionally, similar to skeletal muscle cells, where GLUT4 also translocates due to muscle contractions, in primary rat hippocampal neurons, action potential firing also triggers the insertion of GLUT4 into the plasma membrane, especially in axonal nerve terminals. These data attribute to hippocampal GLUT4 protein the same functional role it plays in insulin-sensitive classic tissues, positioning this transporter as a key player in glucose utilization in the hippocampus. Thus, two transporters are fundamental in glucose transport and therefore for the maintenance of hippocampal cognitive functions; GLUT1, which is responsible for glucose transport across the BBB, supplying glucose to the brain interstice, and GLUT4, which regulates glucose influx into neurons. The latter is crucial in insulin-stimulated peripheral glucose uptake, playing a fundamental role in the metabolic homeostasis of hippocampal neurons in physiological and pathological conditions resulting from neuronal homeostasis disruption [76] (Figure 1).

## 5. Cerebral Energy Metabolism and Steroid Hormone Receptors

It is a fact that estrogens are the master regulator that acts through a network of receptors, ensuring an effective cerebral response by regulating energy metabolism through the coordination of transcriptional and signaling pathways [81]. Estrogens, mainly 17 β estradiol (E2), exert important modulatory functions in the CNS through genomic and non-genomic mechanisms. The sources of E2 for the brain include circulating E2 or testosterone secreted from peripheral tissues and that quickly accesses the CNS and E2 synthesized in neurons and glial cells. ERα and ERβ are the two types of estrogen receptors (ER) that mediate the actions of E2. These receptors act as both membrane-associated proteins that trigger rapid non-genomic effects and transcription factors that mediate the genomic effects of E2. Guanine protein-coupled receptors, such as G protein-coupled ER-1 (GPER1, occasionally referred to as GPR30), also mediate some of these quick effects [82].

**Figure 2:**
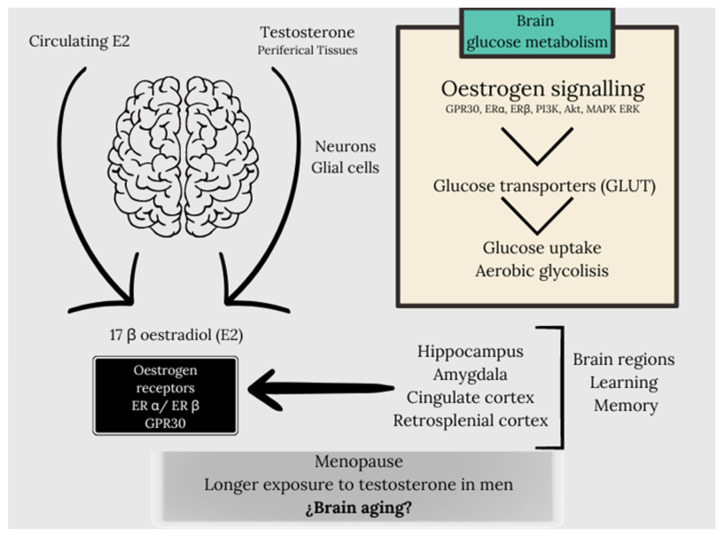
The sources of E2 for the brain include circulating E2 or testosterone secreted from peripheral tissues and that quickly accesses the CNS and E2 synthesized in neurons and glial cells. The effects of E2 are mediated by 2 types of estrogen receptors (ER): ERα and ERβ [82]. Estrogen signaling supports and sustains glucose metabolism in the brain by regulating the expression of glucose transporters, resulting in increased glucose uptake, stimulating aerobic glycolysis. Estrogens regulate energy metabolism in the brain through estrogen receptors, GPER, ER-α and ER-β, activation of PI3K and Akt and MAPK-ERK signaling pathways. In fact, in brain regions linked to learning and memory, such as the hippocampus, the amygdala, the cingulate cortex and the retrosplenial cortex, estrogen receptors are present [92]. In this context, we need to consider whether the process of brain ageing affects men and women equally, taking into account the discrepancy in estrogen and testosterone exposure in both sexes, for example in the case of the menopausal process in the case of women or the longer exposure to testosterone in the case of men.

Studies developed by Cooke et al. (2017) reviewed the implication of estrogens in male brain physiology, considering that local aromatization of testosterone to estradiol is necessary for the development of the male brain, being the effect of testosterone indirect on it. In fact, in the absence of testosterone, undifferentiated brains develop as females. It also establishes that the normal differentiation of the male brain is susceptible to being altered by perinatal exposure to endocrine disruptors (e.g., BPA) [83,84]. Thus, cerebral sexual dimorphism is indirectly controlled by testosterone after local conversion to E2, although other genes of the sex chromosomes could also affect this process. In relation to the steroid receptors that are expressed in the male brain, both aromatase and ERα, Erβ, and GPER have been described [85,86]. The locally produced E2 is considered a cerebral neurosteroid [87] and its effects on the brain in the case of men are mediated by the mentioned receptors, as well as its neuroprotective function of estradiol [88]. In the case of women, the linkage of estrogens in cerebral energy metabolism becomes especially relevant in a vital transition state for women such as menopause. In fact, in women after menopause, those who receive estrogen replacement therapy have a different pattern of brain metabolism than women who do not receive estrogen therapy. However, these results should be taken with caution as there is an important relationship with the time along the perimenopause at which the treatment is administered [89]. In this context, the analysis of 18F-FDGPET has demonstrated indicators of hypometabolism in regions of the brain necessary for learning and memory. In fact, hippocampal hypometabolism, after a 2-year observation period, was evident in women during menopause, both in the parahippocampal gyrus and the temporal lobe area, the medial prefrontal cortex, and the posterior cingulate cortex, and estrogen replacement therapy prevented hypometabolism in each of these brain regions and preserved memory function [90,91,92]. In this sense, it has been established that the metabolism of glucose in the brain is fundamental for neurological function and that evidence of hypometabolism is evident several decades before the diagnosis of neurodegenerative diseases such as Alzheimer’s disease [93,94,95]. Estrogen signaling supports and sustains glucose metabolism in the brain by regulating the expression of glucose transporters, resulting in increased glucose uptake, stimulating aerobic glycolysis. Estrogens regulate energy metabolism in the brain through estrogen receptors, GPER, ER-α, and ER-β, activation of PI3K and Akt, and MAPK-ERK signaling pathways. In fact, in brain regions linked to learning and memory, such as the hippocampus, the amygdala, the cingulate cortex, and the retrosplenial cortex, estrogen receptors are present [96]. At this point, we must ask ourselves whether the effect of estrogens on the process of brain aging affects men and women in the same way, since in the case of men, the absence of periods of changes like menopause for women, as well as a longer exposure to testosterone in the case of men, would make us presuppose different aging processes, linked to the exposure of sex hormones (Figure 2).

In this context, we must note that sex hormones such as androgens and estrogens are powerful modulators of adult neurogenesis in the hippocampus. Estrogens modulate neurogenesis in women, but to a lesser extent in men [97], while androgens modulate neurogenesis in males [98,99], but it is not known if they modulate neurogenesis in women [100]. An interesting review developed by Arevalo et al. [88] highlighted estradiol as a neuroprotector in male and female brain and established that the synthesis of brain-derived estradiol regulates synaptic plasticity, adult neurogenesis, reproductive and aggressive behavior, pain processing, affect, and cognition. It is revealing that they put on stage aromatase, an enzyme that generates estradiol, which, under pathological conditions, increases in neurons and is induced de novo in astrocytes as an endogenous neuroprotective mechanism. Thus, decreased brain aromatase activity would increase gliosis and promote neurodegeneration after brain injury.

**Figure 3:**
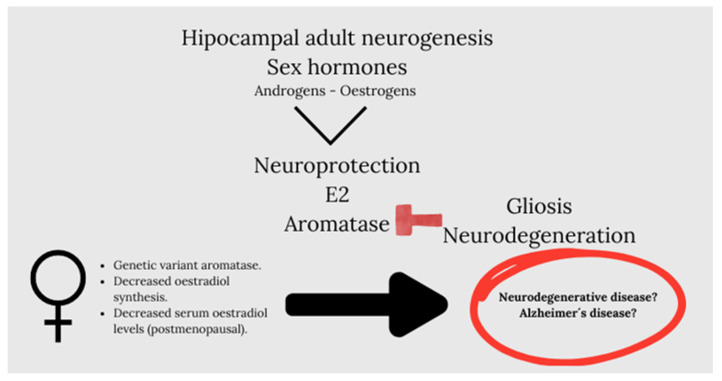
Sex hormones such as androgens and estrogens are potent modulators of adult neurogenesis in the hippocampus. Arevalo et al., highlights estradiol as a neuroprotector in male and female brain. Also, aromatase, an enzyme that generates estradiol, under pathological conditions, increases in neurons and is induced de novo in astrocytes as an endogenous neuroprotective mechanism. Thus, the inhibition or silencing of cerebral aromatase increases gliosis and neurodegeneration. Genetic variants of aromatase may cause a decrease in the synthesis of estradiol in the brain that together with a decrease in serum levels of estradiol in postmenopausal women, or serum levels of testosterone in older men, may increase the risk of developing neurodegenerative diseases.

In this regard, aromatase would be an important neuroprotective molecule in humans, which is suggested by the existence of genetic variants of the enzyme that confer an increased risk of Alzheimer’s disease [101,102,103]. These genetic variants of aromatase may cause a decrease in the synthesis of estradiol in the brain, which, together with a decrease in serum levels of estradiol in postmenopausal women or serum levels of testosterone in older men, may increase the risk of developing neurodegenerative diseases (Figure 3).

Interestingly, the expression of aromatase increases in the astrocytes of the human prefrontal cortex in the late stages of Alzheimer’s disease, a phenomenon that has been interpreted as part of a rescue program [104]. In the human brain, aromatase mRNA, aromatase protein, and aromatase activity have been detected in the hippocampus, the amygdala, the preoptic area, the hypothalamus, and the neocortex [105,106,107]. Initially, it was thought that the function of cerebral estradiol was restricted to the regulation of reproductive behavior and neuroendocrine processes in the hypothalamus [108]. However, locally produced E2 also protects the brain against a variety of neurological and neurodegenerative disorders, including Alzheimer’s disease (AD) [109,110]. In fact, studies developed by Prange Kiel et al. [111] showed the presence of aromatase in sections of the hippocampus of postmortem human brain tissue, in all regions of the cornu ammonis (CA1, CA3, and CA4) and in the dentate gyrus (DG). Most of the cells positive for aromatase were neurons, and according to the location and size of the neurons, it was concluded that the neurons positive for aromatase in the cornu ammonis and DG were pyramidal cells and granular cells, respectively, with a small percentage of non-neuronal cells [88]. On the other hand, a study carried out by Kakimoto et al. [112], conducted with a sample size of 963 cognitively normal humans, addresses the brain changes in both sexes throughout normal aging. They observe a reduction in gray matter volumes predominantly lateral associated with age, as already described earlier [113], as well as significant specific differences by sex in the progression and pattern of reduction of various areas. Thus, a rapid decrease was observed from the age of 60 in men, while in women a gradual decrease was observed from the age of 40. This difference was linked to hormonal events such as childbirth or menopause and to social issues in the case of men, such as retirement. As for the changes in brain metabolism related to age, their studies were in agreement with those described by other authors, describing a glucose hypometabolism associated with age in the medial frontal cortex [114], with the peculiarity that they not only observed a change in metabolism but also in volume, since glucose hypometabolism was also associated with brain atrophy associated with age. Studies with PET technique to assess glucose metabolism showed a decrease in glucose metabolism in men and women in the medial parietal cortex and the lateral frontal cortex. In the case of men, it was associated with a visuospatial sensor [115], while in the case of women it was associated with an integrative region of speech processing that included Broca’s area and a complex region of speech processing [116]. According to these authors, these metabolic differences decreased as age advanced, maintaining differences only in the male parietal prefrontal cortex and female ventrolateral cortex. Already in studies developed in the last century, [117] showed that the morphological characteristics of the brain in humans seem to be sensitive to the effects of age and sex, as we have mentioned earlier, showing, at least according to the data reviewed, that the woman’s brain is more sensitive to these factors, specifically in relation to menopause, where neuronal levels of glucose transporters decrease, which would coincide with the appearance of cerebral hypometabolism [118]. The brain would adapt to this decrease in the availability of glucose by increasing the dependence on ketone bodies as an alternative fuel to generate acetyl-CoA necessary to enter the TCA cycle and, ultimately, generate ATP through complexes of mitochondrial redox transporters. The depletion of ketone bodies derived from hepatic lipid metabolism can lead to the metabolism of fatty acids derived from the brain to generate ketone bodies through oxidation processes in glial cells [119]. We must bear in mind that as Brinton et al. [81] concludes reproductive senescence, that is, menopause, is driven by the ovaries but also by the brain. In fact, the gonadotropin-releasing hormone (GnRH) stimulates the synthesis of E2 in the ovary but also activates the synthesis of E2 in the hippocampus [111,120,121].

## 6. Alteration of Glucose Metabolism: Insulin

In addition to endogenous brain estrogens, circulating estrogens, and aromatase activity, there is another molecule directly involved in cerebral glucose metabolism: insulin.

It should be noted that the effects of insulin in the hippocampus may not exactly coincide with those observed in other parts of the body. In fact, some of its effects are mechanically impossible in the hippocampus; for example, GLUT4 is expressed in neurons [74,122] and glycogen, on the contrary, is only produced in astrocytes [123], so it is unlikely that insulin affects the conversion of glucose to glycogen through GLUT4-mediated glucose uptake in the brain. In general, brain insulin signaling and GLUT4 regulation may differ from those of peripheral tissues. However, it is true that hyperinsulinemia and insulin resistance have significant effects on cognitive impairment and play an important role in AD, being more prevalent in women [124]. In fact, hyperglycemia is a possible risk factor for the development of AD, as well as low insulin response, i.e., insulin resistance.

The possible mediating mechanisms of insulin action come from studies showing a binding relationship between T2DM and the development of AD and showing that AD patients have a reduction in insulin signaling in the hippocampus along with brain hypometabolism and beta-amyloid (Aβ) accumulation [71,125,126,127,128].

Under physiological conditions, insulin binding to its receptor (IR), which is autophosphorylated at Tyr residues and consequently promotes the phosphorylation of its substrate, insulin receptor substrate 1 (IRS1), activates the signaling pathway. Once activated, IRS1 functions as a scaffold protein, leading to the PI3K/Akt axis activation, which is essential for linking upstream effectors (IR and IRS1) with downstream proteins that mediate insulin neurotrophic outcomes. PI3K/Akt axis activation is regulated by the PTEN phosphatase, which reduces the levels of PIP3 required for Akt activation and increases PKCζ expression. Akt promotes the phosphorylation of several targets, including GSK3β and AS160. Furthermore, PKCζ and AS160 are responsible for translocating GLUT4-containing vesicles to the plasma membrane to manage glucose uptake.

Furthermore, Akt stimulates the upregulation of HKII, which is a pivotal enzyme involved in glucose metabolism and thus energy production. During the development of cerebral insulin resistance, alterations in several proteins linked to glucose metabolism have been described. Specifically, the brain insulin resistance phenomenon is associated with reduced levels of IR protein and increased levels of inhibitory phosphorylation of IRS1, which are responsible for the uncoupling between IR and IRS1. As a result, despite that insulin binds to IR, reduced activation of its downstream effectors was described [129]. Therefore, the inhibition of IRS1 is responsible for the uncoupling between IR and IRS1.

The scientific literature undoubtedly links the development of cerebral insulin resistance and hippocampal affection with the risk of developing Alzheimer’s disease [130,131,132]. In fact, Alzheimer’s disease has already been characterized as type 3 diabetes [133,134].

Data provided by Tramutola et al. [129] suggest the existence of defects in the mechanisms responsible for the translocation of GLUT4 to the plasma membrane. Although most glucose uptake in neurons occurs through GLUT3, insulin-regulated GLUT4 is also co-expressed with GLUT3 in brain regions related to cognitive behavior [135]. Insulin activation induces the translocation of GLUT4 to the neuronal cell membrane through an Akt-dependent mechanism [74,136] and is believed to improve glucose flow to neurons during periods of high metabolic demand, such as learning [137].

### 6.1. GLUT4 as an Insulin-Estradiol Mediator

However, along with GLUT4, the vesicles also contain other proteins, some of which are involved in the regulation of memory processes, such as insulin-like growth factor 2 (IGF2) receptors (IGF2R), insulin-regulated aminopeptidase (IRAP), and the Ras GTPase activator-like protein IQGAP1 [138,139,140]. Therefore, the recruitment of GLUT4 to the cell surface will simultaneously recruit these proteins (and vice versa), so that the increase in GLUT4 in the plasma membrane could be a correlation with the cognitive effects of insulin, associated with the peptides with which it is co-transported. The presence of IRAP in GSVs can promote GLUT4 protein stability and regulate GLUT4 compartmentalization and recycling from endosomes to GSVs after its translocation and subsequent endocytosis [141].

The GLUT4 translocation pathway shows points of connection with the action pathways induced through ER α and β, specifically in relation to glucose metabolism through PI3k and Akt. Thus, the binding of estradiol to membrane-associated α and β-ER activates numerous signaling cascades, many of which are linked to neuroprotection. It also activates the insulin-like growth factor 1 (IGF1) receptor (IGF1R), which mediates neuroprotective signals through the transcriptional increase in IGF1 and induces the interaction of the ER receptor with the p85 catalytic subunit of PI3K, composed of this and the p110 unit, allowing the formation of a complex formed by ER, IGF1R, and the components of the IGF1R signaling pathway such as insulin receptor substrate IRS1, PI3K, Akt, and GSK3. Through this pathway, estradiol downregulates Tau phosphorylation [88].

**Figure 4:**
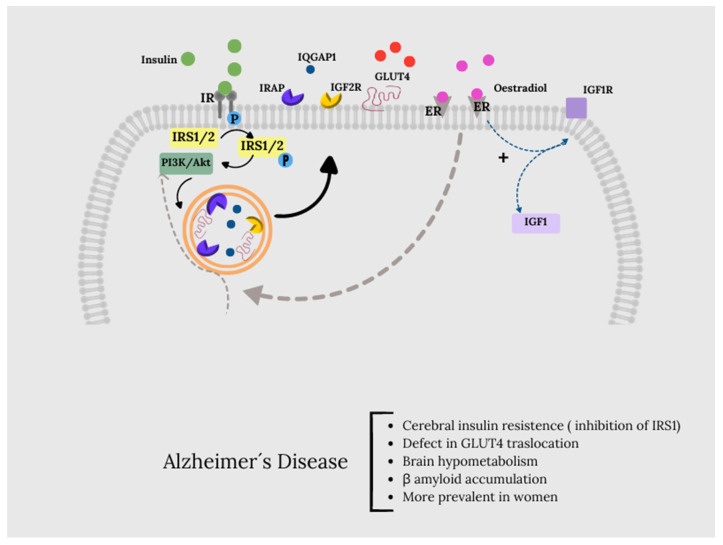
Under physiological conditions, insulin binding to its receptor (IR), which is auto phosphorylated at Tyr residues and consequently promotes the phosphorylation of its substrate, insulin receptor substrate 1 (IRS1), activates the signaling pathway. Once activated, IRS1 functions as a scaffold protein, leading to the PI3K/Akt axis activation, which is essential for linking upstream effectors (IR and IRS1) with downstream proteins that mediate insulin neurotrophic outcomes. Activation of the PI3K/Akt axis is regulated by the phosphatase PTEN, which reduces PIP3 levels required for Akt activation as well as for increasing the expression of PKCζ. Akt promotes the phosphorylation of several targets, among which are GSK3β (on Ser9, inhibitory site) and AS160 (on Thr642, activating site). This latter, together with PKCζ, is responsible for translocating GLUT4-containing vesicles to the plasma membrane to mediate glucose uptake. Along with GLUT4, the vesicles also contain other proteins, some of which are involved in the regulation of memory processes, such as insulin-like growth factor 2 (IGF2) receptors (IGF2R), insulin-regulated aminopeptidase (IRAP), and the Ras GTPase activator-like protein IQGAP1 [138,139,140]. Therefore, the recruitment of GLUT4 to the cell surface will simultaneously recruit these proteins (and vice versa), so that the increase in GLUT4 in the plasma membrane could be correlated with the cognitive effects of insulin, associated with the peptides with which it is co-transported. The GLUT4 translocation pathway shows points of connection with the action pathways induced through ER α and β, specifically about glucose metabolism through PI3k and Akt, the binding of estradiol to membrane-associated α and β-ER activates numerous signaling cascades, many of which are linked to neuroprotection. It also activates the insulin-like growth factor 1 (IGF1) receptor (IGF1R), which mediates neuroprotective signals through the transcriptional increase of IGF1 [88].

In this context, the study of postmortem brains with AD and mild cognitive impairment showed clear indications of brain insulin resistance, i.e., reduced IR and increased serine (inhibitory) phosphorylation of IRS1, particularly in the hippocampus, cortex, and hypothalamus [129,142]. In this sense, although there are few studies related to the relationship between IRS1 and brain estrogens, this connection could indeed occur through the IRS1/PI3K and Akt molecules and ERα and β (Figure 4).

### 6.2. Role of GLUT4 in Hippocampal Cognitive Processes: Importance of IRAP

Hippocampal cognitive processes and glucose metabolism are intimately linked processes and connected through insulin. Insulin is a key player in the cognitive processes of the hippocampus: specific blockade of endogenous intrahippocampal insulin significantly impairs spatial working memory, while physiological doses administered to the hippocampus improve it. The alteration of glucose supply to neurons because of dysregulation of the insulin-sensitive glucose transporter GLUT4, authors such as Mc Nay and Pearson propose as a unifying mechanism that explains, at least in part, the comorbidity between type II diabetes and AD [130,143].

GLUT4 is the main glucose transporter in muscle and adipose tissue and plays a crucial role in neuronal carbohydrate metabolism. Insulin activates within minutes to mobilize GLUT4 from intracellular reserves and controls the number of transporters on the cell surface to regulate glucose absorption in these tissues. One of the main current treatments for type II diabetes and/or insulin resistance is metformin, which increases the insulin sensitivity of tissues by increasing GLUT4 expression [144].

At the beginning of this century, the presence of the GLUT4 transporter in the mouse hippocampus along with the insulin-regulated protein (IRAP) was described [139]. The results shown by Chai et al. suggest that, in the hippocampus, where IRAP and GLUT4 colocalize in the same vesicles, there is an activity-dependent glucose uptake mechanism. This system can be modulated by IRAP ligands, such as angiotensin IV, and depends on both IRAP and GLUT4. Therefore, the discovery of IRAP expressed in intracellular vesicles of the neuronal soma, where GLUT4 is also localized, in brain regions involved in cognitive function, as a binding site for memory-facilitating peptides such as Ang IV, would represent the description of a new function for this enzyme in a murine model [145,146].

Thus, it has been shown that glucose flow through GLUT4 is necessary for cognitive improvement by hippocampal AngIV, suggesting that IRAP is not an alternative to GluT4 as the transducer of the cognitive effects of insulin [147].

In this context, the results found by Ismail et al. [148] show a molecular relationship between cholesterol, brain glucose, and the brain renin–angiotensin system, all of which are affected in some neurodegenerative diseases. Specifically, they observed that 27-hydroxycholesterol (27OH) was able to curb the ability of AngIV to stimulate glucose uptake and inhibit the catalytic activity of IRAP. Recently, the 27OH has been defined as an endogenous selective estrogen receptor modulator (SERM) [149], with activities in atherosclerosis, osteoporosis, breast and prostate cancers, and neurodegenerative diseases. Although the blood-brain barrier is impermeable to cholesterol and, therefore, a cholesterol-rich diet does not modify intracranial cholesterol concentrations, 27OH is a slightly more polar metabolite than cholesterol and can cross the blood-brain barrier. In this sense, a recent study by Brooks et al. on late-onset AD described the reduction of ERα and an elevation of ERβ in association with a high level of cholesterol/27OH. The hippocampal regions of AD patients have a similar pattern of decreased ERα and increased ERβ.

## 7. Conclusions

Brain development characteristics in both sexes lead us to consider pathological differences at the brain level for each sex, along with the described plasticity of hippocampal tissue and the distinct pattern of receptors present in this tissue, making it susceptible to different ligands and defining its response to them. Therefore, we could deduce from the review conducted that a comprehensive understanding of the functional, anatomical, and functional differences in brain tissue, particularly the hippocampus, as well as the energy metabolism where hormones like insulin and estradiol play a crucial role, along with glucose and its transporters and related molecules such as insulin-regulated aminopeptidase, would provide us with valuable information for comprehending and subsequently studying pathologies resulting ultimately from the aforementioned processes and associated with sex, such as neurodegenerative pathologies, specifically Alzheimer’s disease.
